# Analysis of Microchannel Heat Sink of Silicon Material with Right Triangular Groove on Sidewall of Passage

**DOI:** 10.3390/ma15197020

**Published:** 2022-10-10

**Authors:** Surojit Saha, Tabish Alam, Md Irfanul Haque Siddiqui, Mukesh Kumar, Masood Ashraf Ali, Naveen Kumar Gupta, Dan Dobrotă

**Affiliations:** 1 Department of Mechanical Engineering, Indian Institute of Engineering Science and Technology, Howrah 711103, India; 2 CSIR—Central Building Research Institute, Roorkee 247667, India; 3 Mechanical Engineering Department, King Saud University, Riyadh 11421, Saudi Arabia; 4 Department of Industrial Engineering, College of Engineering, Prince Sattam Bin Abdulaziz University, Al-Kharj 16273, Saudi Arabia; 5 Institute of Engineering and Technology, GLA University, Mathura 281406, India; 6 Department of Industrial Engineering and Management, Faculty of Engineering, Lucian Blaga University of Sibiu, 550024 Sibiu, Romania

**Keywords:** silicon material, right triangular groove angles, Nusselt number, microchannel, heat sink

## Abstract

Microchannel heat sink (MCHS) is a promising solution for removing the excess heat from an electronic component such as a microprocessor, electronic chip, etc. In order to increase the heat removal rate, the design of MCHS plays a vital role, and can avoid damaging heat-sensitive components. Therefore, the passage of the MCHS has been designed with a periodic right triangular groove in the flow passage. The motivation for this form of groove shape is taken from heat transfer enhancement techniques used in solar air heaters. In this paper, a numerical study of this new design of microchannel passage is presented. The microchannel design has five variable groove angles, ranging from 15° to 75°. Computational fluid dynamics (CFD) is used to simulate this unique microchannel. Based on the Navier–Stokes and energy equations, a 3D model of the microchannel heat sink was built, discretized, and laminar numerical solutions for heat transfer, pressure drop, and thermohydraulic performance were derived. It was found that Nusselt number and thermo-hydraulic performance are superior in the microchannel with a 15° groove angle. In addition, thermohydraulic performance parameters (THPP) were evaluated and discussed. THPP values were found to be more than unity for a designed microchannel that had all angles except 75°, which confirm that the proposed design of the microchannel is a viable solution for thermal management.

## 1. Introduction

These days, the use of electronic gadgets is increasing due to their wide applications in telecommunication, automobile, aerospace, computing, robot, medical, and chemical industries, etc. [1,2,3,4]. With the advancement of technologies, these gadgets have been developed to integrate very tiny components such as electronic chips, microcontrollers, microprocessors, transistors, etc. These components are very sensitive to heat and can be damaged if heat is not dissipated. Therefore, the thermal management of such components is of utmost necessity for their proper functioning and long life. It has become necessary to explore the technique of extracting the excess heat from these electronic components. The primary technique of heat removal by means of forced convection using air (as heat transfer fluid) is the most common practice [5,6,7,8,9]. However, these techniques are restricted in those cases where very high heat flux densities, high temperature, and space constraints are dominant. Therefore, microchannel heat sinks are appealing to researchers because they have several advantages, such as a higher heat dissipation rate, compactness, and direct substrate.

Numerous studies on thermal and hydraulic characteristics of microchannel heat sinks (MCHS) have been carried out since Tuckerman and Pease’s [10] breakthrough. Most of the studies employed the laminar forced convection fluid flow in a simple microchannel with parallel plates [11]. Other studies include the usage of nanofluids as heat transfer fluid, which are employed in the MCHS as their thermo-physical properties can be altered as per requirements [12]. However, in mathematical models, the nanofluids are governed by two-phase flow and their mathematical model does not predict the fluid phenomenon accurately. However, single-phase flow in the microchannel is accurately predicted by traditional Stokes and Poiseuille flow theories [13].

The most common and attractive techniques for well-performing MCHS are the passive techniques, which exploit the geometrical modification to improve the heat transfer coefficient. Geometrical parameters play an important role in deciding the final geometry such as the orientation of the cavities, pitch, depth, and tip length of MCHS. By adjusting these geometrical parameters, the cavity of the triangular shape changed to trapezoidal, and eventually rectangular. These modifications are responsible for improving the Nusselt number up to 51.29% [14].

The basic concept for exploiting the geometrical modification is flow disruptions, reducing the thermal interfacial area, or elevating the velocity distribution along the walls of the heat sink [15]. The following studies have focused on these passive techniques. Wang et al. [10] showed the enhancement of the thermal performance of MCHS with a series of trapezoidal grooves. Shui et al. [16] showed the analysis of MCHS of tree-like branching wherein dimple roughnesses was exploited. Their finding showed that dimple roughnesses were beneficial for increasing the average heat transfer rate. Micro inserts are also alternatives to geometrical modification in existing MCHS. Adding inserts can accelerate the fluid flow, resulting in strengthening the heat transfer capabilities [17]. Geometrical parameters, including the shape of the cross-sectional passage such as rectangular, trapezoidal and triangular, have been studied. The cross-sectional of the passage is governed by hydraulic diameter MCHS with the smallest hydraulic diameter outperforming the other heat sinks in terms of pressure drop and friction factor [18].

A review of MCHS design was presented, which revealed that improper design could result in increased pumping power and, as a result, an imbalance of thermal characteristics [19]. Therefore, the proper design of the microchannel is necessary for optimum performance. In this regard, researchers around the world are exploring various designs of the passage of MCHSs to develop optimum thermal management. The passage of MCHS has been explored, such as channel shape in the wavy form [20] and tapered form [21]. Additionally, the cross-section of the passage in the following shapes—circular [22], wedge [23], rectangular [24], triangular [25] and square [26]—have been exploited. Further, the effect of surface modification in a passage in the form of fins [27], ribs [28], fillets [29], dimples [30], sinusoidal wavy [31] and periodic expansion–constriction [32] have been explored and investigated. These geometrical modifications have shown significant improvement in the heat dissipation rate.

In the aforementioned discussion, it was revealed that various techniques have been exploited wherein geometrical modifications are widely exploited along with the use of nanofluids. In addition, the triangular groove in the passage has been exploited [33,34], but the right triangular groove has not been studied so far. Therefore, keeping eyes on the literature gap, the right triangular groove in the passage has been proposed for further study. Since water is more accessible and secure than using nanofluids improperly in a microchannel heat sink, it is more prevalent as a heat transfer fluid [35]. So, it is imperative to exploit the right triangular groove with water as heat transfer fluid. In this regard, this study aims to exploit the right triangular groves in flow passage in combination with water as a heat transfer fluid. The geometry of a microchannel in the form of the right triangular groove in the wall of the passage of the heat sink is thus numerically simulated in this work. The effect of the angle of right triangular grooves has been studied, which has not yet been carried out.

## 2. Computational Model of Microchannel Heat Sink

### 2.1. Computaional Domain

**Figure 1:**
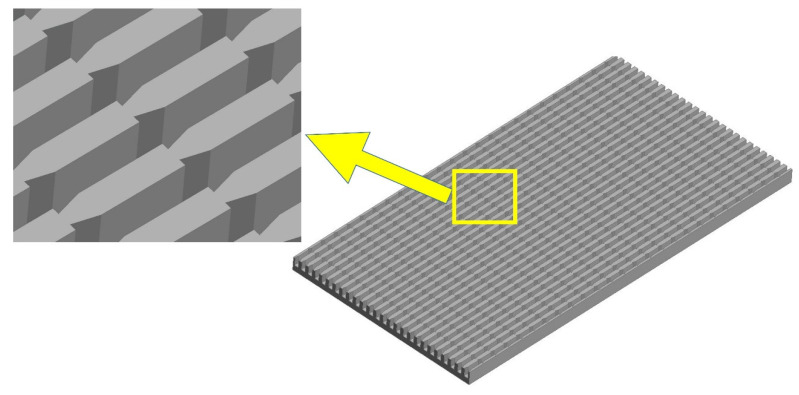
Microchannel Heat Sink (MCHS).

**Figure 2:**
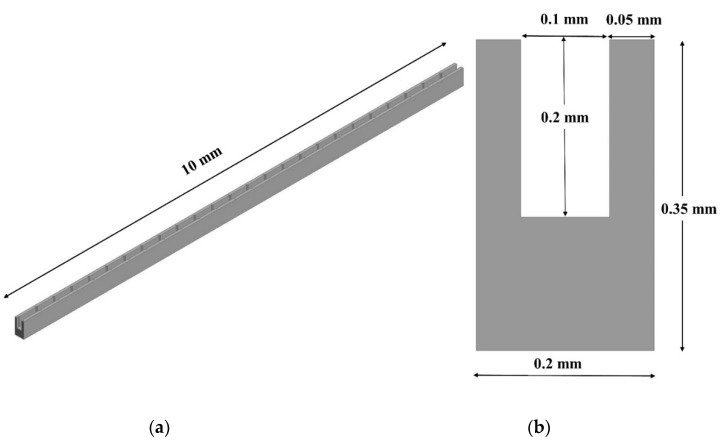
3-D Computational Domain (**a**) isometric view, (**b**) cross-sectional view.

**Figure 3:**
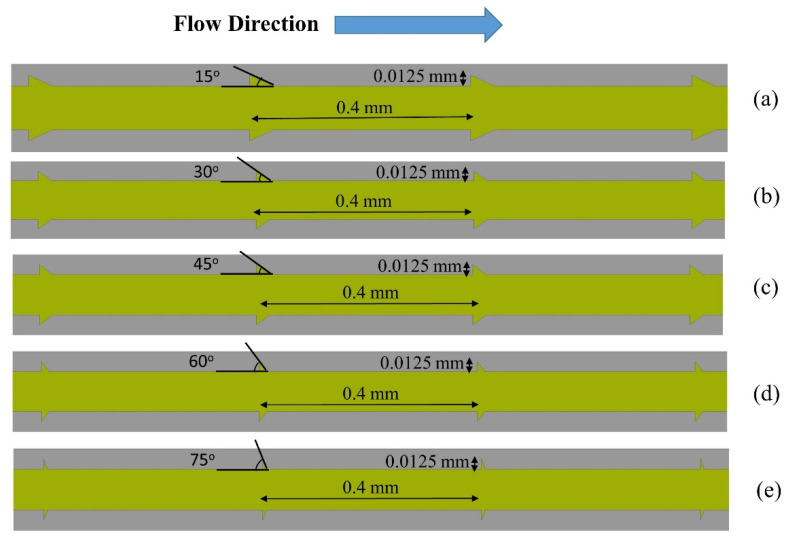
Fluid and Microchannel Domain with different right triangular groves (Top view) (**a**) 15^o^ (**b**) 30° (**c**) 45° (**d**) 60° and (**e**) 75°.

The microchannel heat sink and fluid’s computational domain were created using Ansys Workbench’s design module. The domain was three-dimensional, non-isothermal in a steady state, and used single-phase fluid (water). The micro-channel heat sink was composed of 50 straight channels with a rectangular cross-section and 25 pairs of triangular grooves in each channel as shown in Figure 1. CFD simulation of this would have required enormous computing resources and would definitely be lengthy. Therefore, one channel of MCHS having a length of 10 mm, a height of 0.35, and a width of 0.2 mm was numerically simulated for all cases as suggested by Chai et al. [34]. The pitch of the right triangular groove in the passage of MCHS was 0.4 mm based on the study presented by Chai et al. [32]. Since the thickness of the passage wall was 0.05 mm, the groove depth was taken as half of the passage wall, i.e., 0.025 mm. The domain of microchannel and fluid with the dimensions are shown in Figure 2. The base interface between the fluid domain and the microchannel was planar and the side walls had triangular grooves with varying angles from 15° to 75°; however, edge thickness was not varied and was kept constant at 0.025 mm. The pitch of triangular grooves was 0.4 mm in all cases as suggested by Chai et al. [32]. The variation in groove angles on the wall of the flow passage of the microchannel is illustrated in Figure 3.

### 2.2. Conservation Equations

This subsection presents mathematical governing equations. For steady-state conditions, single-phase, and laminar flow, the following equations were solved. Incompressible water was used as a heat transfer medium in this study, flowing through a microchannel.

In all expressions, the subscripts “s” and “f” refer to solid (silicon) and fluid (coolant), respectively.

The conservation of mass equation or the equation of continuity is:

∇·(𝜌_f_·u) = 0. (1)

The momentum conservation equation is:

(u·∇)·𝜌_f_·u= −∇p+μ_f_·∇^2^u, (2)

where u represents velocity [m/s], p represents pressure [Pa], 𝜌_f_ represents density [kg/m^3^] and μ_f_ represents dynamic viscosity [N·s/m^2^] of the coolant.

The energy equation is given by:

(3) $$u\cdot \text{∇}T=\frac{k_{f}}{\left(\rho_{f}\cdot \mathrm{Cp}\right)\;\left(\nabla^{2}\right)T},$$

where k_f_ represents the thermal conductivity [W/m·K], Cp represents the specific heat [J/kg·K] and T represents the temperature [K].

The equation of energy for solid microchannel (silicon) is given by:

k_s_∇^2^·T = 0, (4)

where k_s_ represents the thermal conductivity of the silicon [W/m·K].

### 2.3. Thermo-Physical Properties of Microchannel Heat Sink Material and Heat Transfer Medium

The following computation domains were used in the simulations, i.e., microchannel and heat transfer fluid, wherein silicon was used as the microchannel material and water was used as the heat transfer medium. The following tables (Table 1 and Table 2) list the thermo-physical properties of silicon (as microchannel heat sink’s material) and water (as a heat transmission medium), respectively, which are assumed for running the simulations.

### 2.4. Computational Procedure and Boundary Conditions

For velocity–pressure coupling in the multi-grid solution process, the SIMPLE (Semi Implicit Method for Pressure Linked Equations) algorithm was used. The solutions were obtained by an iterative process using convergence criteria of 1.0 × 10^−6^ for the velocity component and momentum, while convergence criteria were 1.0 × 10^−9^ for energy. The boundary and initial conditions were set before the iterations. The inlet velocity of the fluid was given as per the select Reynolds number in the range of 100–900. The outlet of the flow was considered an outflow in the atmosphere; therefore, the pressure outlet condition was given with a zero pressure gauge. The base of the microchannel’s bottom wall was subjected to a uniform heat flux of 1.0 × 10^6^ W/m^2^. The side walls of the MCHS were given as symmetry to simulate 50 adjacent passages of MCHS.

### 2.5. Grid Independent Study

**Figure 4:**
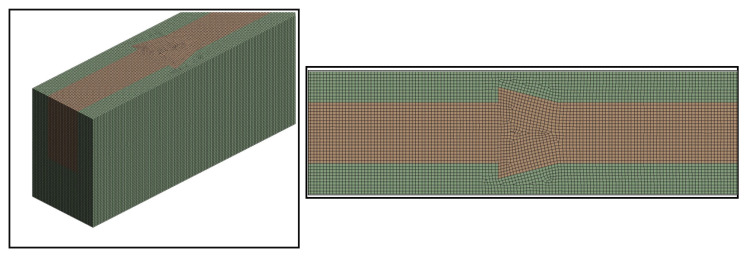
The meshing of Fluid and Microchannel Domain.

The Ansys Workbench design modeler was used to build the respective computation domains for single microchannel and fluid domains. Thereafter, a multi-zoned structured mesh with a very fine mesh size was created, as illustrated in Figure 4. Several test simulations were run to make sure the results would be independent of the mesh. This was achieved by grid-independent test wherein several simulations run by changing the numbers of nodes in the ranges of 284,675–1,427,663. Table 3 shows the number of nodes, number of elements, values of Nusselt number, and corresponding error in two consecutive Nusselt numbers. It can be clearly seen that the error changes from 0.233% to 0.029% when the node increases from 284,675 to 1,659,354. Further, an increase in node number does not show a significant error in the Nusselt number, therefore, 1,659,354 nodes were considered for all further simulations.

### 2.6. Data Acquisition

Numerical solutions of MCHS that had a right triangular groove on the passage of sidewalls were used to gather the data. Data reduction was carried out to present useful results in terms of the friction factor, Nusselt number, and flow pumping power. To present these results, the following parameters were evaluated.

The Reynolds number is written as:

(5) $$Re=\frac{\rho_{f}\;\mu_{m}\;D_{h}}{\mu_{f}}.$$

The hydraulic diameter is defined as:

(6) $$D_{h}=\frac{2\;W\;H}{W+H}.$$

The average friction factor is given as:

(7) $$\bar{f}=\frac{\Delta p\;D_{h}}{2\;\rho_{f}\;L\;u_{m}^{2}\;}.$$

The average convective heat transfer coefficient is given as:

(8) $$\bar{h}=\frac{q\;A_{q}}{A_{c}\;\left({\bar{T}}_{c}-{\bar{T}}_{f}\right)}.$$

Conjugated area avg temperature is given as:

(9) $$\bar{T_{c}}=\frac{\int T\;dA}{\int dA}.$$

Mass avg temperature of the coolant is given as:

(10) $$\bar{T_{f}}=\frac{\int T\;\rho_{f}\;dV}{\int \rho_{f}\;dV}.$$

The average Nusselt number is formulated as:

(11) $$\bar{Nu}=\frac{\bar{h\;}D_{h}}{k_{f}}.$$

Pumping of coolant in microchannel heat sink is defined as;

(12) $$P=\Delta p\;u_{m}\cdot A_{cs}.$$

### 2.7. Model Validation

**Figure 5:**
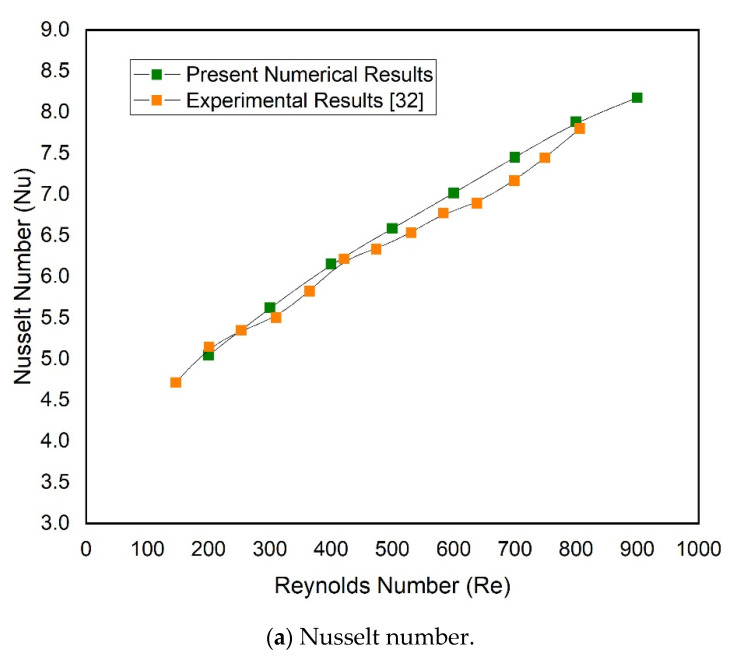

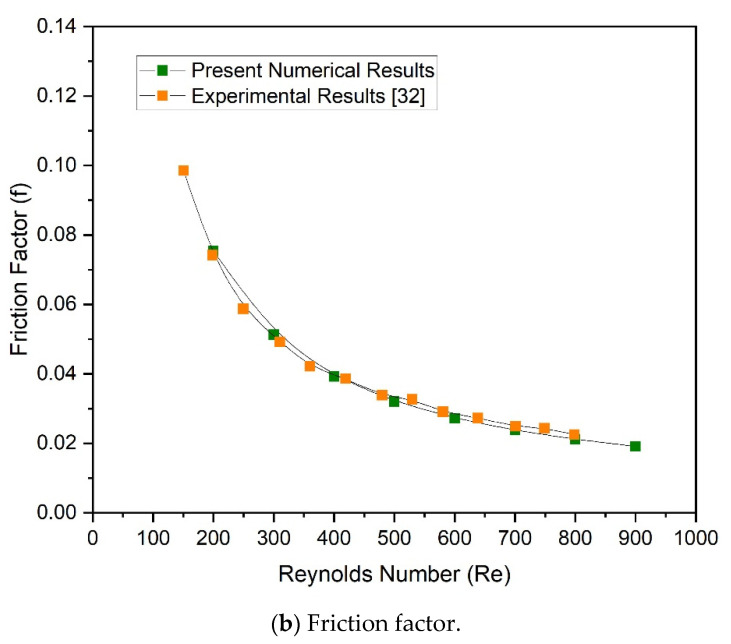
Validation of present numerical results of (**a**) Nusselt number and (**b**) friction factor with experimental results of Nusselt number and friction factor by Chai et al. [32].

In this sub-section, the numerical results of conventional smooth MCHS without a right triangular groove on the sidewall of passage are compared with well-known experimental results of similar MCHS, presented by Chai et al. [31]. Figure 5 shows the present results of Nusselt number and friction factor for a conventional microchannel along with those experimental results of Nusselt number and friction factor that have been plotted. These graphs show that the friction factor and Nusselt number values are in good agreement with the respective experimental values of friction factor and Nusselt number. The current numerical solution was found to be reliable; therefore, further simulations were carried out using the same numerical model.

## 3. Result and Discussion

The microchannel with pairs of triangular grooves on the sidewalls was numerically simulated using CFD with Reynolds numbers ranging from 100 to 900. The velocity and pressure contours inside the flow domain, the temperature distribution of fluid and microchannel domain at mid-plane, and temperature distribution at the micro channel’s base are discussed in the following subsections.

### 3.1. Velocity Distribution

**Figure 6:**
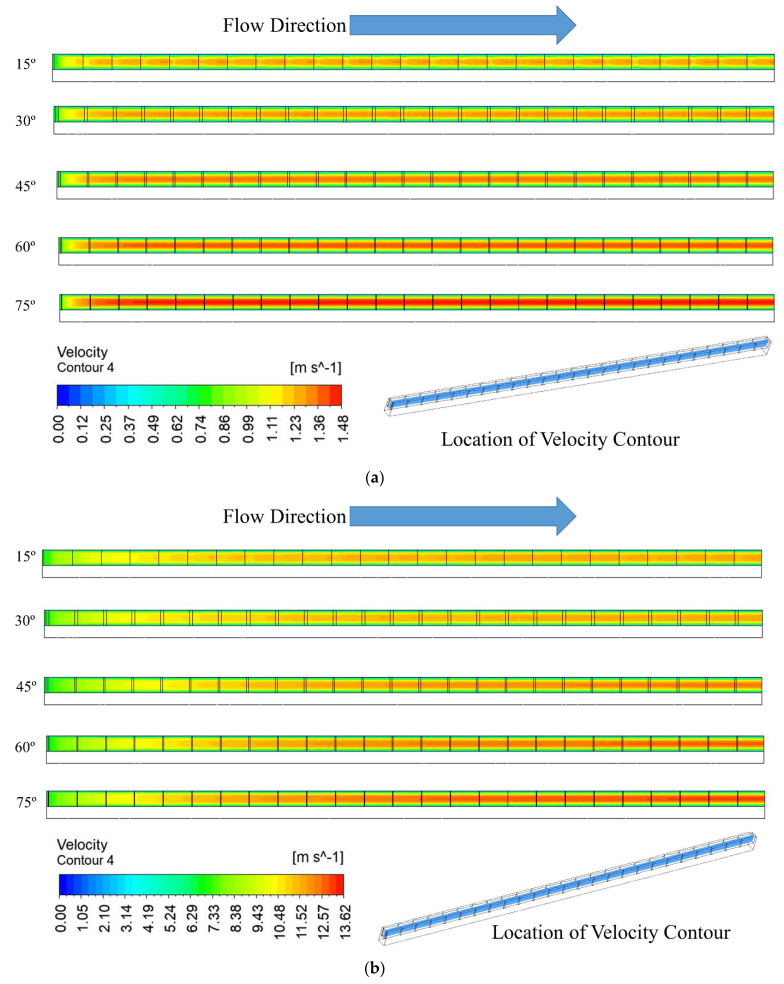
Velocity contour at the midplane of fluid domain for (**a**) Re = 100, (**b**) Re = 900.

Figure 6 compares the velocity distribution in the fluid flow field at various right triangular groove angles for two extreme Reynolds numbers (100 and 900). At both Reynolds numbers, i.e., 100 and 900, it is evident that maximum velocity slightly rises with increasing groove angle. This occurs as a result of flow, which is not disturbed by a large right triangular groove angle, i.e., 75°. However, flow is disturbed due to a low right triangular groove angle of 15°. Higher disturbance in the flow due to low groove angle contributes to a higher heat dissipation rate. Similarly, maximum velocity increases slightly with an increase in the right triangular groove angle at a Reynolds number of 900. In addition to this, velocity changes significantly for all right triangular groove angles when the Reynolds number changes from 100 to 900.

### 3.2. Pressure Drop Distribution

**Figure 7:**
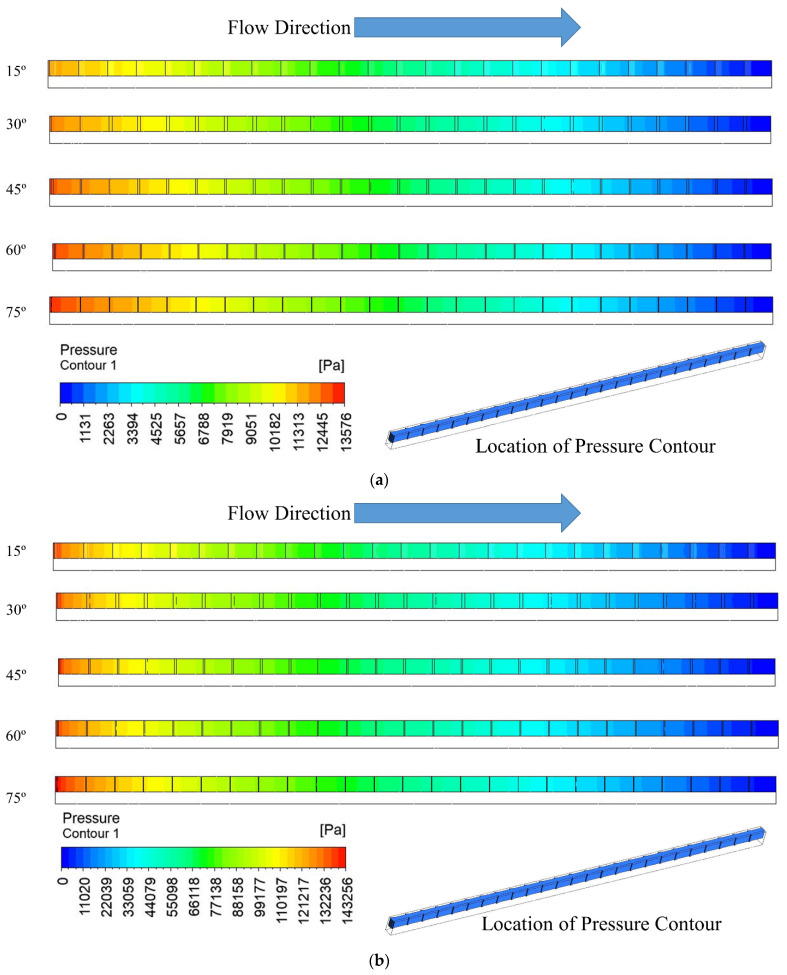
Pressure contour of fluid domain for (**a**) Re = 100, (**b**) Re = 900.

Fluid domain pressure contours in the passage of microchannel when operating at different Reynolds numbers of 100 and 900 are presented in Figure 7. The illustration shows the presence of a pressure drop in the fluid domain for all angles along the micro channel’s length. At a Reynolds number of 100, a microchannel with a groove angle of 15° has the lowest pressure drop and pressure drop along the length of the microchannel increases slightly with an increase in groove angle from 15° to 75°. A similar trend in pressure drop has been found when the Reynolds number changes from 100 to 900. However, a considerable amount of pressure drop increases when the Reynolds number varies from 100 to 900 for all right triangular groove angles. This change in pattern happens because the viscosity of water at higher Reynolds numbers offers significant restriction.

### 3.3. Temperature Distribution

**Figure 8:**
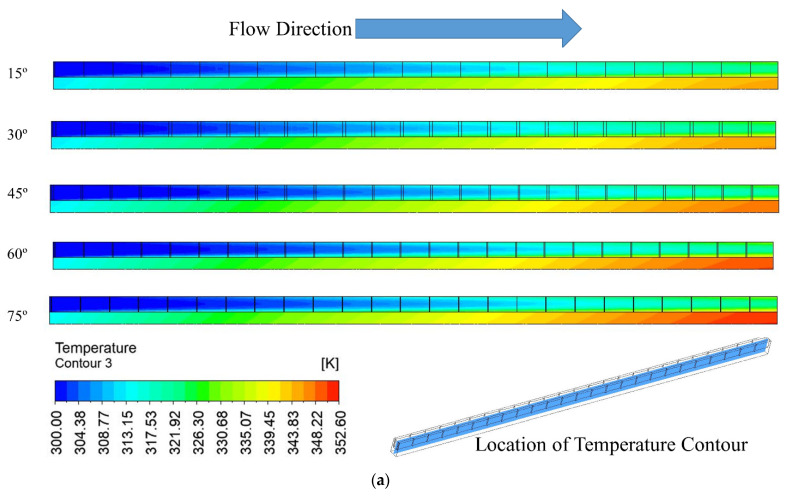

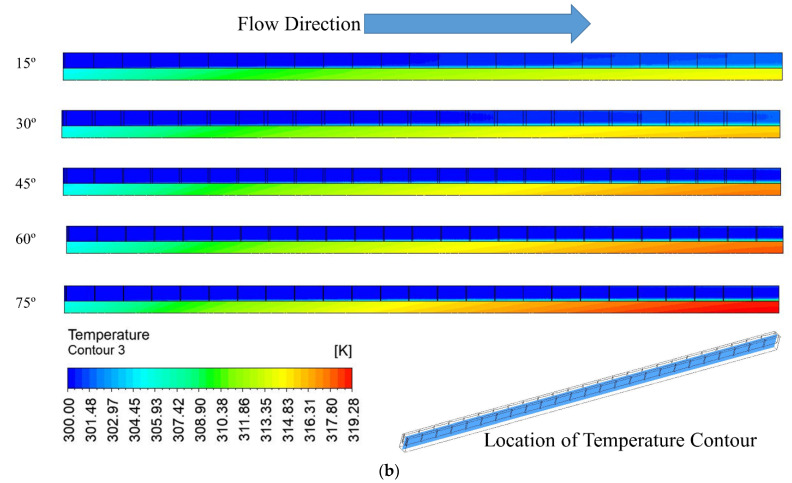
Temperature contour at the mid plane of fluid and microchannel domain for (**a**) Re = 100, (**b**) Re = 900.

The temperature distribution for the various groove angles at two Reynolds numbers of 100 and 900 are shown in Figure 8 on a plane placed at the midpoint of the fluid domain and microchannel heat sink. The maximum temperature is displayed on the bottom wall, where the electrical chips are installed. The coolant flow temperature rises with the flow direction, reaching its highest temperature at the outlet. The fluid temperature rises from the intake to the outflow at all angles and for both values of the Reynolds number. For a particular Reynolds number, maximum temperature slightly increases with an increase in right triangular groove angle changes from 15° to 75°. Because of the lower temperature, a lower heat dissipation rate is induced. The Reynolds number changes from 100 to 900, which contributes to an increase in maximum temperature; this is because higher Reynolds numbers extract more heat due to suppression of the viscous laminar layer.

**Figure 9:**
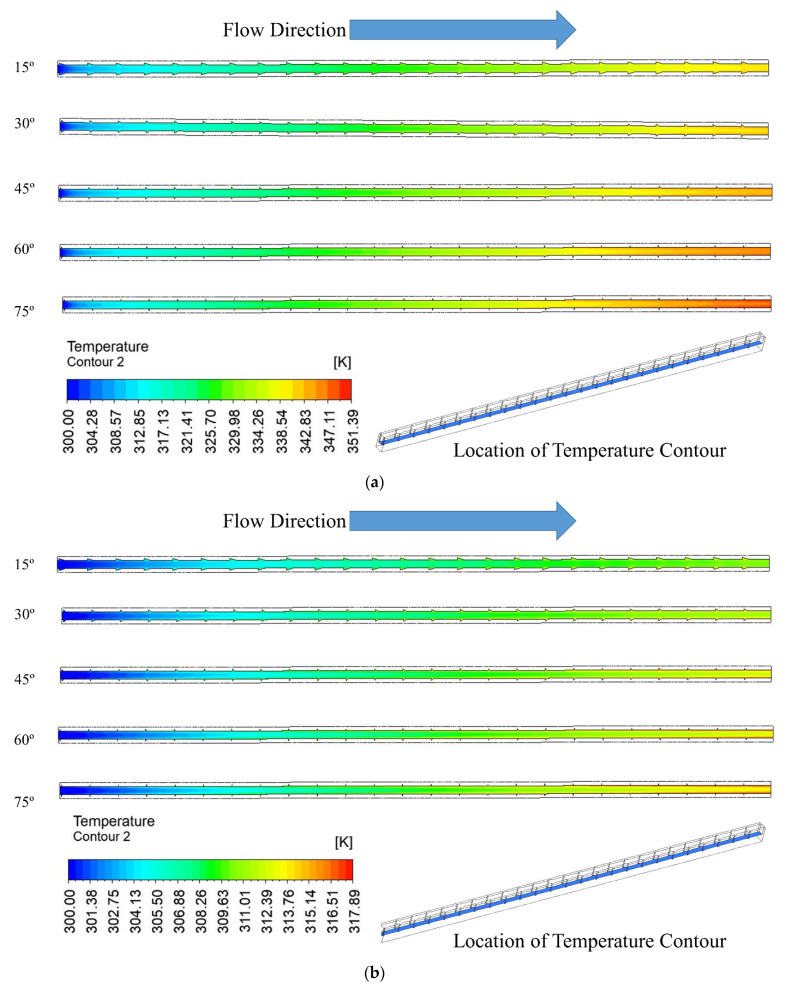
Temperature contour at the base of microchannel passage for (**a**) Re = 100, (**b**) Re = 900.

Figure 9 shows the base of the microchannel wherein the temperature contours due to various groove angles at two values of Reynolds numbers (i.e., 100 and 900) are presented. It can be seen from the contours that the temperature distribution over the base of the microchannel is similar at both Reynolds numbers. However, the maximum temperature increases slightly with an increase in the right triangular groove angle from 15° to 75° at both Reynolds numbers. This increase in base temperature indicates the decrease in heat dissipation rate from the microchannel. Similarly, for the same groove angle, the maximum temperature of the base of the microchannel decreases significantly, which is attributed to a higher heat dissipation rate when the Reynolds number changes from 100 to 900.

### 3.4. Performance Analysis

Different right triangular groove angles of the sidewall of the microchannel heat sink that can affect heat transfer and fluid flow characteristics were investigated to quantify the performance of the microchannel heat sink in terms of Nusselt number, friction factor, pumping power, and thermohydraulic performance. These parameters of heat transfer and friction characteristics of a microchannel with varying right triangle groove angles are presented and discussed in the following sections.

**Figure 10:**
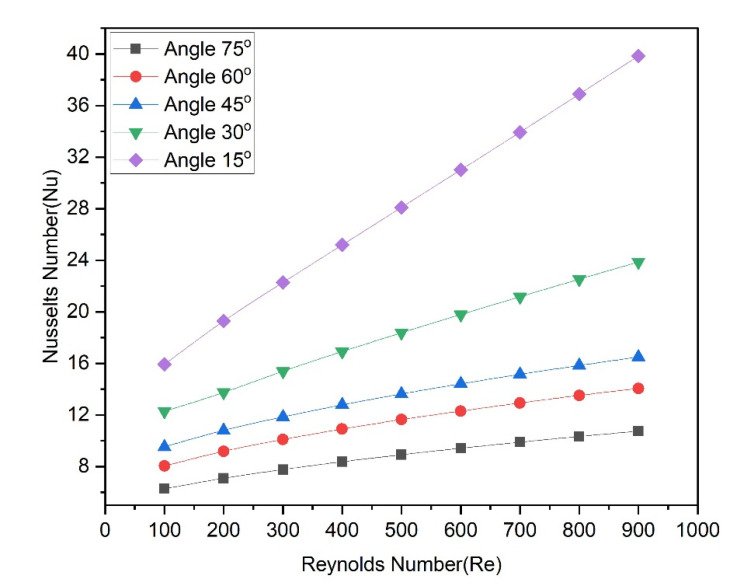
Variation of Nusselt number with Reynolds number at various angles.

**Figure 11:**
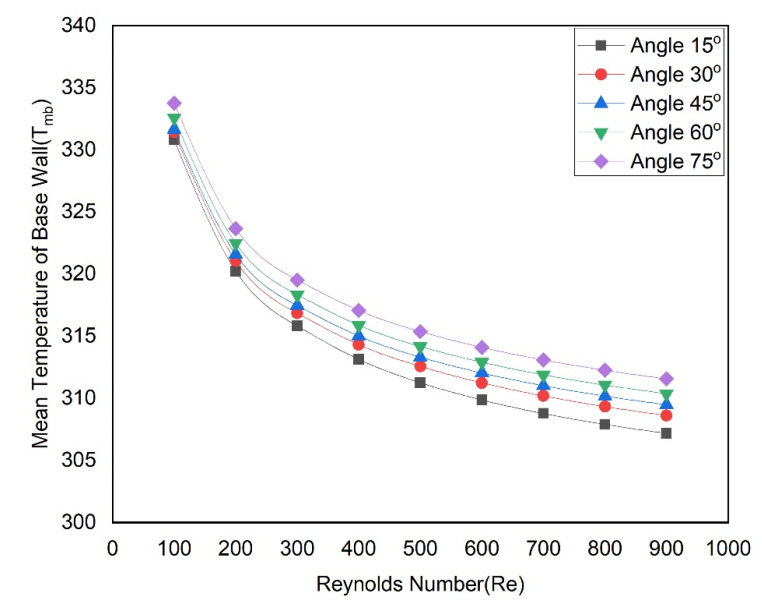
Variation of temperature at the base of microchannel passage with Reynolds numbers at various angles.

Figure 10 shows the variation of the Nusselt number with Reynolds numbers as groove angles are altered. From all angles, it is discovered that Nusselt number trends are continuously rising concerning Reynolds number trends. Nusselt numbers are higher for the 15° groove angle and lower for the 75° groove angle. The Nusselt numbers vary in the following ranges of 15.93–39.84, 12.30–23.86, 9.54–16.51, 8.05–14.07, and 6.27–10.76 for groove angles of 15°, 30°, 45°, 60° and 75°, respectively. Plots of the base temperature of the microchannel, as shown in Figure 11, can explain this trend in the Nusselt number. For all angles, it is evident that, when the Reynolds number rises, the base temperature decreases. At lower Reynolds numbers, the temperature declines fast and, at higher Reynolds numbers, the temperature becomes asymptotically. The lowest base temperatures are obtained for all Reynolds numbers when the groove angle is 15°, while the highest base temperatures are found when the groove angle is 75°. The higher microchannel base temperature is attributed to a lower Nusselt number in the case of the 75° groove angle while the lower microchannel base temperature is attributed to a higher Nusselt number in the case of 15° groove angle.

**Figure 12:**
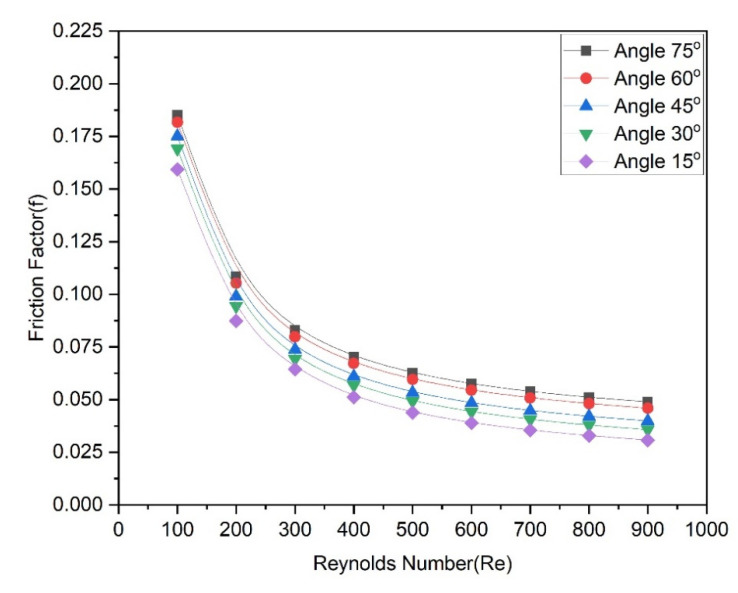
Variation of friction factor with Reynolds number at various angles.

Figure 12 illustrates that the friction factor of the heat transfer medium fluid changes as the Reynolds number changes with different groove angles. For all angles, the friction factor continuously decreases as the Reynolds number rises. It is observed that the rate of decrease in the friction factor at low Reynolds numbers is faster than the rate at higher Reynolds numbers. It is also found that the friction factor with a lower groove angle (15°) has the lowest values while the friction factor with a lower groove angle (75°) has the highest values. In other words, friction factor values increase with the increase in groove angle from 15° to 75°, which indicates that the groove angle strongly affects the friction factor. To demonstrate this, Figure 12 is prepared to show the pumping power as a function of the Reynolds number at various groove angles.

**Figure 13:**
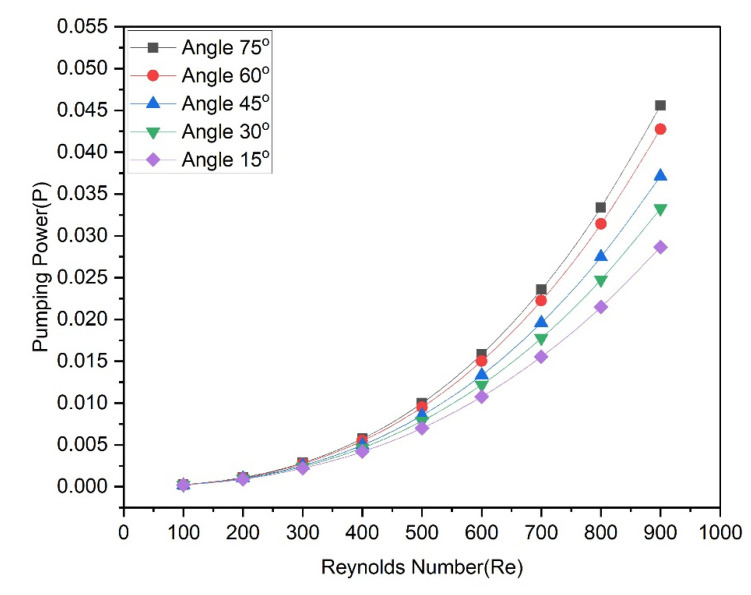
Variation of pumping power with Reynolds number at various angles.

It can be seen from Figure 13 that pumping power increases slightly with an increase in the lower range of Reynolds numbers, while significant pumping power increases with the increase in the higher range of Reynolds numbers for all groove angles. It is also found that the highest values of pumping power are attained at the groove angle of 75°, while the lowest values of pumping power are achieved at a groove angle of 15°.

The enhancement in Nusselt number and friction factor, occurring as a result of the right triangular groove in the passage of MCHS in comparison to the smooth passage of MCHS, are expressed in terms of enhancement factor, expressed as:

Enhancement factor in Nusselt number = Nu/Nu_s_ (13)

Enhancement factor in friction factor = f/f_s_. (14)

The values of the enhancement factor in Nusselt number and friction factor due to right triangular groove in the passage of MCHS are evaluated and presented in Table 4. It can be found that the maximum enhancement factor in the Nusselt number is found at 4.87 in the case of a 15° right triangular groove; however, the minimum enhancement factor in the Nusselt number is found at 1.31 in the case of a 75° right triangular groove. Similarly, the maximum enhancement factor in the friction factor is found at 2.56 in the case of a 75° right triangular groove; however, the minimum enhancement factor in Nusselt number is found at 1.08 in the case of a 15° right triangular groove. This enhancement factor in Nusselt number and friction confirm the benefit of exploiting the right triangular grooves in the passage of MCHS.

As aforementioned, the data of the Nusselt number and friction factor power alone do not decide the best configuration of the right triangular groove angle. It is required to take into account both friction factor and Nusselt number at the same time to demonstrate the overall thermohydraulic performance of the microchannel with a distinct groove angle. Therefore, the hydraulic performance parameter (THPP) was used to demonstrate how the groove angle affects the overall functionality of the microchannel heat sink as given below by Equation (15). It is stated that if the THPP value for a design is greater than one, then the overall result will invariably be successful regardless of the individual values of friction factor and Nusselt number.

(15) η=Nu/Nusf/fs1/3.

**Figure 14:**
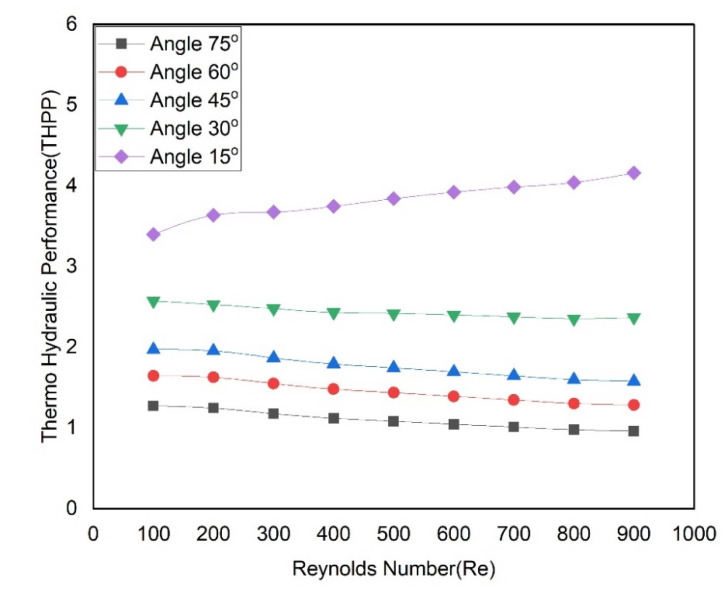
Variation of Thermo-hydraulic performance parameters (η) vs. with Reynolds numbers at various angles.

The values of THPP are calculated and presented as a function of the Reynolds number at various groove angles and are shown in Figure 14. THPP values of a microchannel having distinct groove angles do not have a regular trend. THPP values increase with an increase in Reynolds number in the case of a microchannel having a groove angle of 15° while values of THPP slightly decrease with an increase in Reynolds number for other groove angles. Maximum values of THPP have been found in the range of 3.40–4.16 in the case of a groove angle of 15°, while minimum values of THPP have been found in the range of 1.27–0.96 in the case of a groove angle of 75°. Additionally, it is observed that THPP values of a microchannel having all angles except a 75° angle are found to be more than 1. Therefore, a microchannel with a right triangular groove from 15° to 60° on sidewalls would have an advantage over the flat ones.

## 4. Conclusions

Right triangular grooves on the sidewalls of the microchannel passage design have been proposed in this study. Five various groove angles from 15° to 75° were considered for analysis. Ansys Fluent’s CFD code was used to simulate a microchannel utilizing water as the heat transfer medium. The following findings were made using mathematically projected results:

- The right triangular groove angle on the sidewalls of the microchannel strongly affects the heat dissipation rate;
- It is observed that the Nusselt number and friction factor strongly depend on the Reynolds number. The Nusselt numbers varied in the following ranges of 15.93–39.84, 12.30–23.86, 9.54–16.51, 8.05–14.07, and 6.27–10.76 for 15°, 30°, 45°, 60° and 75° groove angles, respectively;
- A 15° groove angle provided the highest augmentation in Nusselt number augmentation, while a 75° angle provided the highest augmentation in friction factor;
- The enhancement factor is the Nusselt number and the friction factor was evaluated. The maximum enhancement factor in Nusselt number and friction factor were found at 4.87 and 2.59 in the case of 15° and 75° right triangular grooves, respectively. However, the minimum enhancement factor in Nusselt number and friction factor were found at 1.31 and 1.08 in the case of 75° and 15° right triangular grooves, respectively;
- THPP values were found to be more than unity for a designed microchannel that had all angles except 75°, which confirms that the proposed design of the microchannel is a viable solution for thermal management;
- The TPHH of the microchannel varies from 3.40 to 4.16, 2.57 to 2.37, 1.97 to 1.58, 1.64 to 1.28, and 1.27 to 0.96 at corresponding groove angles of 15°, 30°, 45°, 60° and 75°, respectively.

This paper presented a numerical study that would help readers to understand the design and development of MCHS. Since this is a numerical study, several assumptions have been made to show the insight view. However, the experimental study can be beneficial for presenting the actual operation of such MCHS. Further, nanofluid is an alternative to increasing performance; however, several problematic issues have been associated with the usage of nanofluid in MCHS. Therefore, appropriate nanofluids tailored to specific MCHS should be developed in the future.

## Figures and Tables

**Table 1:** Thermo-physical characteristics of water.

Fluid	Density [kg/m^3^]	Dynamic Viscosity μ [Pa·s]	Specific Heat Cp [J/kg·K]
Water	998.2	0.001	4182

**Table 2:** Heat sink material’s thermo-physical characteristics.

Material	Density 𝜌_s_ [kg/m^3^]	Thermal Conductivity k_s_ [W/m·K]	Specific Heat Cp_s_ [J/kg·K]	Young’s Modulus E_s_ [GPa]	Thermal Expansion ℘ [1/K]	Poisson’s Ratio α
Silicon	2329	130	700	170 × 10^9^	2.6 × 10^−6^	0.28

**Table 3:** Grid independent test.

Sl. No.	Nodes	Element	Nusselt Number (Nu)	Percentage Variation in Nu (%)
1	284,675	226,746	30.2083	-
2	622,064	519,112	24.48375	0.233
3	1,032,831	873,456	21.60522	0.133
4	1,248,404	1,058,027	19.8444	0.088
5	1,659,354	1,427,663	19.28312	0.029

**Table 4:** Enhancement in Nusselt number and friction factor.

Reynolds Number	Enhancement Factor in Nusselt Number (Nu/Nus)					Enhancement Factor in Friction Factor (f/f)				
	15°	30°	45°	60°	75°	15°	30°	45°	60°	75°
100	3.49	2.7	2.09	1.76	1.37	1.08	1.15	1.19	1.23	1.26
200	3.82	2.73	2.14	1.82	1.41	1.16	1.25	1.31	1.4	1.44
300	3.96	2.74	2.11	1.8	1.38	1.25	1.35	1.44	1.56	1.62
400	4.09	2.75	2.08	1.77	1.36	1.3	1.45	1.55	1.71	1.79
500	4.27	2.79	2.07	1.77	1.35	1.37	1.54	1.67	1.86	1.95
600	4.42	2.82	2.06	1.75	1.34	1.43	1.62	1.78	2.00	2.11
700	4.55	2.84	2.03	1.74	1.33	1.49	1.71	1.88	2.14	2.26
800	4.68	2.86	2.01	1.71	1.31	1.55	1.79	1.99	2.27	2.41
900	4.87	2.92	2.02	1.72	1.32	1.61	1.87	2.08	2.4	2.56

## Data Availability

Not applicable.

## Nomenclature

A_c_	Conjugated Area (i.e.: the area of the solid-fluid interface) (m^2^)
A_q_	Heated Area (i.e., silicon base area) (m^2^)
D_h_	Hydraulic Diameter of the microchannel (m)
f	Friction Factor
L	Characteristics Length (m)
h	Heat transfer coefficient
H	Height of microchannel (m)
K	Thermal Conductivity (W/m·°K)
Nu	Nusselt Number
p	Pressure (Pa)
q	Heat Flux that applied to the bottom wall of the micro heat sink (W/m^2^)
Re	Reynolds Number
T_c_	Conjugated Area Average Temperature (K)
T_f_	Mass-Average Temperature of the Coolant in the Microchannel (K)
u	Velocity (m/s)
W	Width of microchannel (m)
∆p	Pressure Drop
P	Pumping power (w)
q	Heat Flux (W/m^2^)
**Greek letters**	
μ	Dynamic Viscosity (N·s/m^2^)
η	Thermo hydraulic performance parameters (THPP)
ρ	Density (kg/m^3^)
**Subscripts**	
b	Microchannel base
f	Fluid (coolant)
s	Solid (silicon) or Conventional
